# Complexities of contemporary urban planning in local government in the City of Polokwane, Limpopo province

**DOI:** 10.4102/jamba.v15i1.1326

**Published:** 2023-02-17

**Authors:** Ngoako J. Mokoele

**Affiliations:** 1 Department of Development Planning and Management, Faculty of Management and Law, University of Limpopo, Polokwane, South Africa

**Keywords:** climate change, colonial planning system, complexity theory, greenhouse gases, urbanisation

## Abstract

Urbanisation is characterised by the high consumption of nonrenewable resources and being resource-intensive in meeting the energy demands of the growing urban population. The growth compels the efficient management of urbanisation to mitigate climate change. The inability to effectively plan and manage urbanisation will result in high consumption of nonrenewable resources, greenhouse gas (GHG) emissions and pollution, which contribute to the intensification of climate change. Complexity theory as a theoretical framework asserts that the management of urbanisation is both complex and nonlinear. This means that the management of urbanisation cannot be done by reducing the system into individual components. The study adopted both qualitative and quantitative approaches to research. The data were collected from the four areas around the City of Polokwane and officials from the Polokwane Local Municipality. The finding of the study is that the City of Polokwane is still facing multiple challenges such as traffic congestion, lack of community participation, illegal dumping of wastes and a decline in green spaces. Furthermore, the Polokwane Local Municipality has made inroads towards reducing traffic congestion through the implementation of Bus Rapid Transit (BRT) (Leeto la Polokwane). It can be concluded that urbanisation in the City of Polokwane is not effectively planned and managed to address climate change.

**Contribution:**

This article recommends that the Polokwane Local Municipality should implement a solar system plant and generate gas from the increasing level of waste in the City of Polokwane. Furthermore, the Polokwane Local Municipality should transition from operating street lights, office lights and traffic lights with electricity towards the use of solar systems.

## Introduction

Globally, the rate of urbanisation has been unprecedented, which has contributed to the intensification of climate change (UN-Habitat 2011; Mokgotho & Mokoele 2020). Consequently, developed countries have been blamed for the combustion of fossil fuels for the acceleration of the economy with no regard for environmental conservation and protection (United Nations Framework Convention on Climate Change [UNFCCC] 2015). However, the blame game is insignificant in the climate change discourse (Mogano & Mokoele 2019; Mokgotho & Mokoele 2020; Mongala et al. 2019). Some of the major cities in developing countries such as Mumbai, Bangaluru, Lagos and Johannesburg continue to emit greenhouse gases (GHGs) when compared to some of the cities in developed countries (Cobbinah, Erdiaw-Kwasie & Amoateng 2015). South Africa is the only African country counted as one of the top 20 carbon emitters in the world (Cobbinah et al. 2015).

In South Africa, the process of urbanisation took place without proper systems and institutions to effectively plan and manage it (Mokoele & Sebola 2018; Monama, Mokoele & Mokgotho 2022; Ogbazi 2013). Monama et al. (2022) postulate that challenges such as traffic congestion, high consumption of electricity and GHG emissions have overwhelmed the capacity of developing countries to manage urbanisation by applying unreformed planning systems that are inherited from the colonial era. Watson (2009) eloquently states that the complexity of an urban system is rooted in the interaction of economic growth, economic development and social systems. The management of the urban complex system to address urban challenges requires a new methodological approach to urbanisation (Cobbinah & Darkwah 2016). The approaches call for the contextualisation of urban planning philosophy that will serve as enablement for cities to unravel and offer a concerted, yet effective, planning and management paradigm in addressing the aftermath of urbanisation. Closer scrutiny on the urbanisation process with these characters located in a small space is unsustainable.

## Colonial planning and management approaches in post-independence Africa

In Africa, most countries were colonised, and the administration that emerged under the auspices of the colonial administration was discriminative and exploitative towards the nonwhite population (Cobbinah et al. 2015; Cobbinah & Nimingu-Beka 2017). Post-independence in African countries, the remnants of the colonial planning and management ideologies remain unreformed. The use of a colonial top-down planning approach has failed to address urban challenges. Although UN-Habitat (2009) has projected that the global urban population will reach 70% by 2050 (Da Silva et al. 2019), Africa is projected to reach 60%. According to Freire, Lall and Leipziger (2014), based on the current urban population trends, the African urban population is expected to triple in the next 50 years, thus requiring policy frameworks that will help harness the opportunities presented by urbanisation for sustainable development and inclusive growth (Cobbinah & Nimingu-Beka 2017).

The rate of urbanisation has resulted in the:

[*E*]ncroachment on and extinction of public open spaces and ecologically sensitive areas, the pervasiveness of slums communities, unregulated informal economic activities, congestion, flooding, haphazard and unauthorised land development and increasing conversion of traditional land into other uses. (Cobbinah & Nimingu-Beka 2017:389)

In Nigeria, the implementation of the National Urban Development Policy espouses the democratic principles to guide the management of cities to attain sustainable urban development (Ogbazi 2013). This policy drew lessons from the Environmental Planning and Management (EPM) process, which was conceptualised by UN-Habitat (Ogbazi 2013). The policy promotes the participation of the citizen in planning within the city. According to Ekemode (2019), the multiplicity of urban challenges such as traffic congestion and unsustainable use of nonrenewable resources compelled a shift by the Nigerian government to implement the urban renewal strategy, which was meant to upgrade slums, transport infrastructure, drainage systems and disposal systems (Ekemode 2019; Raimi, Adelopo & Yusuf 2018). This urban renewal as a participatory planning approach did not yield intended collaborative planning. The inability of the urban renewal strategy to renew the city through the eradicated informal settlement was based on the lack of a pre-demolition and resettlement scheme or plan (Abgola & Jinadu 1997). The demolition of the informal settlement resulted in the displacement of over 40 000 people and only just over 2000 people were relocated (Abgola & Jinadu 1997; Ekemode 2019). The urban renewal strategy led to the displacement of people who procured the land from the traditional leaders (Ekemode 2019). The displacement of informal settlers resulted in many of them without a place to stay. According to Abgola and Jinadu (1997), the population that relocated to other places such as Ikota Ilasana government estates were found to be in a dire situation. This was because most houses were not completed, they lacked water, sanitation and electricity (Abgola & Jinadu 1997).

Urban planning in African countries such as Zimbabwe and Ghana is inherited from the colonial rule, which largely focused on the establishment of town councils as control centres (Cobbinah & Darkwah 2016; Cobbinah & Nimingu-Beka 2017; Musandu-Nyamayaro 2008). This system was based on a master planning system that was adopted from the British colony as a way to regulate and control growth development in the city. Post-independence in Ghana, Zimbabwe, Nigeria and South Africa, the successive government failed to decentralise planning and management approaches (Cobbinah & Nimingu-Beka 2017) by ensuring participatory urban planning. However, years after Ghana gained independence, the planning approaches remain centralised and insensitive to community aspirations because of the limitations of public engagement in decision-making.

## Rapid and unplanned urbanisation

In developing countries, urbanisation remains unplanned because of the proliferation of urban sprawls, destruction of ecologically sensitive landscape, erection of congestion, increasing consumption of resources and deteriorating urban greenery. Large cities in Ghana such as Accra and Kumasi are characterised by rapid unplanned urbanisation, which resulted in the densification of the urban population and increased the demand for commercial spaces contributing to poor urban planning, weak institutions, lack of enforcement of the planning approaches and political interference (Cobbinah et al. 2015; Cobbinah & Niminga-Beka 2017). According to Korah et al. (2016), Kumasi, which is the second-largest city in Ghana, is characterised by unprecedented urbanisation and unregulated and unplanned development. The densification of the urban population in these Ghanaian cities has overwhelmed the capacity of the urban planner for well-managed urbanisation, which is characterised by less traffic congestion, recycling waste, implementation of an energy mix and reduced electricity consumption to ensure environmental protection.

In South Africa, 28 years since the dawn of democracy and the culmination of apartheid planning frameworks, multifarious urban challenges such as traffic congestion, high waste generation, high electricity consumption and the reduction of urban greenery continue to plague the many cities (Mokgotho & Mokoele 2020; Mokoele & Sebola 2018; Monama et al. 2022). Scholars posited that the inability to manage urbanisation to address urban problems such as traffic congestion, informalities, emissions and high waste production is because of the unreformed colonial planning systems (Cobbinah et al. 2015; Cobbinah & Niminga-Beka 2017; Korah et al. 2016). Despite the successive planning and management systems, the White Paper on Local Government, the Constitution of the Republic of South Africa (1996), *Local Government Municipal Structured Act*, 1998, *Local Government Municipal Systems Act*, 2000 and Integrated Framework of Urban Development (Musandu-Nyamayaro 2008), practically, urban planning remains unreformed and discriminative. This is evidenced by the exclusionary planning system and fragmented governance where citizens remain outside the mainstream of decision-making and planning. According to Musandu-Nyamayaro (2008), South Africa’s legislative framework has been regarded as one of the best in the world. Tsheola, Segage and Ramonyai (2014) argue that the legislative goodness of South Africa should be based on the pragmatic evidence felt by the ordinary populace. Despite the overwhelming affirmation about the goodness of South Africa’s legislative frameworks and systems in managing cities, until it can effectively help resolve the contemporary urban problems and reform the urban landscape, its goodness will remain as a theoretical discourse without any pragmatic evidence to qualify it.

## Urban planning and management praxis in addressing urbanisation

The infrastructure in South Africa, such as road and transport systems, sewage systems and drainage systems, is pragmatic evidence that demonstrates that urban planning has failed to envisage the challenges accompanying the process of urbanisation (Balkaran 2019; Cobbinah & Darkwah 2016; Mapitsa 2019). According to Shen et al. (2013), the challenges associated with rapid urbanisation include the powerlessness of local municipalities to meet the infrastructural needs and enormous municipal services of the growing population while ensuring that the environment is protected. Local municipalities are paralysed by multifarious problems in the cities such as service delivery provision and the lack of capacity (expects) in planning and management of urbanisation. The densification of the urban population fostered the increasing need for huge energy input and resources to maintain the urban metabolism (Morris et al. 2016). The New Urban Agenda through Urban and Territorial Planning reconceptualises the approach to urban planning towards embracing human rights (UN-Habitat 2018), such as the provision of housing, water and sanitation, infrastructure, and land tenure. Furthermore, the New Urban Agenda was aimed at the improving the standard of living through ensuring the availability of adequate food, clothing and shelter (UN-Habitat 2018).

### Urban planning approaches

According to Caldeira and Holston (2015), the creation of urban policy that promotes citizen participation:

[*I*]s one of the best examples of the efforts of citizens to make planning work for democracy and democracy work in the space of everyday to counter entrenched social inequality. (p. 2001)

Complexity theory describes cities as complex systems that are very complex (Cilliers 1998; Poturgali 2013) to plan and manage amid all these changes. The collaborative approach to planning allows the city to dislodge various urban challenges (Caldeira & Holston 2015; Savini, Majoor & Salet 2015). The approach asserts that local government has the responsibility to include all stakeholders, including civil society, communities, business and the private sector, in the planning and management process. This means that planners are no longer the masters of city planning but facilitators and learners in planning philosophy. The collaborative planning approach allows the locals to innovate and participate in planning (Caldeira & Holston 2015; Savini et al. 2015). Innovation and creativity embodied in collaborative planning are attained through the engagement and participation of the locals within municipal planning systems. This is because the local population possesses multiple abilities that are important in planning. Furthermore, urban planning approaches are now embracing human rights in their planning. The human rights–based approach and the right to the city advocate for the empowerment of vulnerable groups of the population. In South Africa, the Integrated Development Plan (IDP) is strategically implemented to foster a collaborative and participatory approach to planning and implementation processes (Mapitsa 2019). Participatory planning is a tool used to overcome the current problems associated with the ever-increasing urban population. The innovative potential that the people in the cities hold remains unharnessed and untapped. Therefore, the innovative power of cities remains an elegant theoretical conception without any pragmatic evidence on the ground. The participatory approach is viewed differently from region to region, focusing on its applicability in an urban context. It should be considered that trans-active, deliberative, communicative and collaborative planning has numerous explanations that offer an important type of public participation in planning, which highlights various approaches in the accomplishment of the shared power (Ogbazi 2013). These descriptions enable the citizenry’s voices to be heard and acknowledged during the formulation of urban policy, hence creating a shared understanding between the citizens and government in planning for urbanisation.

### Urban management approaches

Globally, cities are a complex mixture of human formations that interact with each other through diverse social, environmental, economic and cultural factors (Alnsour 2016; Cilliers 1998; Crawford 2016; Portugali 2011). These interactions do not operate in a vacuum, but their operation takes place under strict policies, social influence, and institutional and legislative frameworks (Alnsour 2016). To ensure proper management of urbanisation, Alnsour (2016) postulates that managing urban population growth occurs through the master plan, zoning ordinances and development plans. In South Africa, the government has promulgated the *Spatial Planning and Land-Use Management Act* (SPLUMA), the Spatial Development Framework (SDF), USDF and IDP as an approach to ensure that the management of urbanisation is efficient and effective to protect the environment and mitigate climate change. The improvement of the quality of life and proper management of urbanisation can profoundly improve environmental sustainability and curb climate change. Management of urbanisation has been enhanced through the technological and cultural transference that was experienced globally (Akhondzadeh-Noughabi 2013). According to Da Silva et al. (2019), cities play an important role in the fight against climate change and other environmental challenges. This takes place through the implementation of new and smart technology to reduce GHG emissions (Balkaran 2019; Da Silva et al. 2019; Oteng-Ababio, Owusu & Asafo 2019). These multiple challenges, such as GHG emissions, declining green places, pollution, traffic congestion and waste, are the embodiment of most cities in developing countries. Polokwane is the economic hub of Limpopo province in South Africa, which is characterised by high temperature, declining annual rainfall patterns and a burgeoning urban population. The rate of urbanisation in Polokwane continues to increase, which contributes to the increasing GHGs, traffic congestion and high waste production.

## Methodology

This article adopted a mixed methods research approach to uncover and explore the reality and existing laws that define the world humanity resides in (Bless et al. 2013). The mixed research approach helped to provide data from the municipal planners and the community to establish the level of collaboration between the municipality and the communities around the City of Polokwane. The target population of the study was selected based on the different socio-economic statuses and locations and differently affected by traffic congestion. The data were collected from Legae la Batho, Emdo Park, Serala View and Flora Park, which are located in Polokwane Local Municipality, Limpopo province. Legae la Batho and Emdo Park are located in the west of the City of Polokwane, which experiences illegal dumping of waste. Serala View and Flora Park are located on the eastern side of the City of Polokwane. These urban areas are located closest to the city. Serala View is a middle-income urban area. The majority of people in Legae la Batho, Flora Park, Serala View and Emdo Park are still economically active, which adds an invaluable contribution to urban planning in an attempt to curb climate change. The study is significant as it unravels the importance of collaboration in ensuring effective urban planning. The stratified sampling design was adopted and simple random sampling was used to sample within the different strata. The number of sampled respondents was 185 from the four communities. In Legae la Batho, 67 respondents were sampled and 71 in Emdo Park, while in Serala View and Flora Park 27 and 20 respondents were sampled, respectively. The qualitative data was collected from officials responsible for IDP and environmental planning, as well as the director of Development and Planning. Structured questionnaires were used to collect quantitative data from the households, while semistructured interview schedules were used to collect qualitative data. Quantitive data was computed and captured on the Statistical Package for the Social Sciences (SPSS). Qualitative data was analysed through thematic analysis.

### Ethical considerations

Ethical approval to conduct this study was obtained from the University of Limpopo Turfloop Research Ethics Committee (reference number: TREC/226/2018:PG).

## Analysis and presentation of findings

The data provided evidence that Polokwane Local Municipality continues to struggle to uproot the top-down planning approach in municipal planning. Thus, the municipality cannot foster a collaborative planning approach. The management of the environment is still a problem because of the decline in green spaces and illegal waste disposal. The data that was presented is from qualitative and quantitative data approaches.

### Public involvement in municipal planning

**FIGURE 1:**
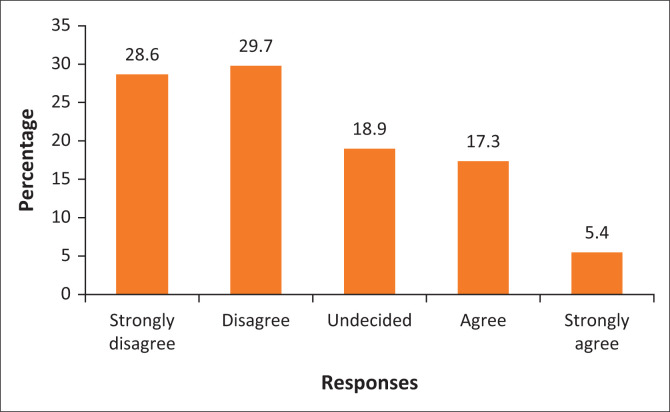
Public involvement in municipal planning.

Figure 1 shows that 28.6% of the respondents strongly disagreed that they were involved in municipal planning while 29.7% disagreed. Only 18.9% of the respondents were undecided on whether they were involved in municipal planning or not. On the other hand, Figure 1 shows that 17.3% of the respondents agreed that the municipality involves the community in planning. A minority (5.4%) of the respondents strongly agreed that they were involved in municipal planning. This means that there is no collaboration between the Polokwane Local Municipality and the communities in planning. An employee in the Development of Water, Sanitation and Forestry stated that:

‘IDP is failing the community in terms of addressing issues that the communities are facing. The IDP is addressing the shopping list. The municipality is not providing the community with feedback after undertaking the IDP processes.’ (Respondent A, Municipal official, Department of Water, Sanitation and Forestry, Interview 15 May 2019)

According to the participant, the frustration of the community is based on the fact that IDP is a strategic municipal planning document, and the IDP guidelines stipulate that people should be at the centre of development. The communities are not provided with feedback on the IDP processes in Polokwane Local Municipality. The finding is that although numerous legislative frameworks stipulate that municipalities should promote public participation, in actuality, communities are left out within municipal planning. This affirms the assertion by scholars such as Tsheola et al. (2014) that municipalities are grappling to promote public participation within municipal planning. This might suggest that there is a shift in the right direction towards the promotion of public participation in municipal planning. Therefore, the lack of public involvement within the municipality derails the potential for collaborative municipal planning and effective management of urbanisation.

### A top-down approach to urban planning

**FIGURE 2:**
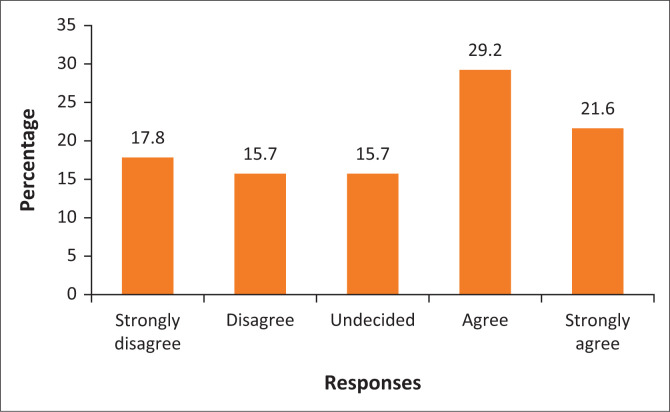
The top-down approach to urban planning.

Figure 2 shows that 17.8% of the respondents strongly disagreed that the Polokwane Local Municipality is still using a top-down approach to urban planning, while only 15.7% disagreed. On the other hand, Figure 2 shows that 29.2% and 21.6% of the respondents agreed and strongly agreed, respectively, that urban planning remains top-down, while only 15.7% remained undecided on this notion. This suggests that many people around the City of Polokwane are excluded from participating in municipal planning. Therefore, this demonstrates that there is no collaborative planning approach within Polokwane Local Municipality. From qualitative data, a key informant from the Polokwane Local Municipality concurred that there is no collaboration by stating that ‘[t]here is a lack of consultation with the communities regarding the plans about the city. The municipality imposes the decisions on the communities’. Literature on urban planning in Africa argues that urban planning remains unreformed from the colonial planning approach. Therefore, the communities around the City of Polokwane perceive urban planning as being unreformed and operating on a top-down approach. An employee in the Capricorn District Municipality stated that:

‘We engage thoroughly with the communities in order to ensure that there is public participation. Again, the municipality engages with the communities so that they can have a full understanding of the plans and development around the municipality.’ (Respondent B, Employee in the Capricorn District Municipality, Interview 15 May 2019)

There is a small proportion of respondents who remained undecided on whether the municipality is still using a top-down approach to urban planning. Evidently, the Polokwane Local Municipality continues to be characterised by a colonialised top-down approach to planning, which is remote from the notion of collaborative planning.

### Green spaces within the City of Polokwane

**FIGURE 3:**
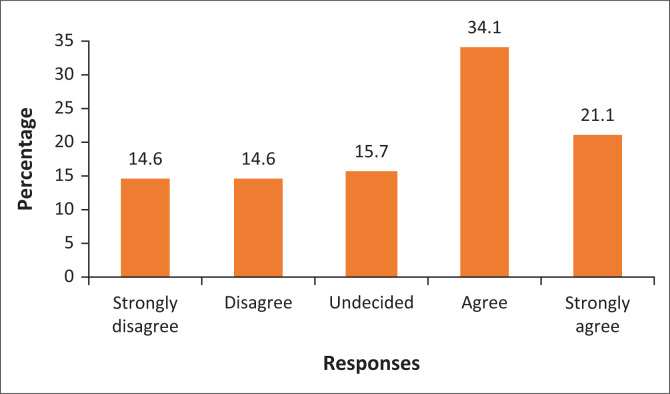
The rate of growth is reducing the number of green spaces around the city.

The 14.6% of the respondents in Figure 3 represent those who strongly disagree and disagree that the growth of the city results in the reduction of green spaces. It is indicated in Figure 3 that only 15.7% were undecided, while 34.1% of the respondents agreed that the level of green spaces has reduced because of the growth of the city. Figure 3 shows that 21.1% of the respondents strongly agreed that the growth of the city reduces the number of green spaces. The majority of the people believed that the green spaces have been reducing as the City of Polokwane continues to grow. From qualitative data, a key informant from the Polokwane Local Municipality stated that ‘[*t*]his is because of an insufficient plantation to attract rainfall during the process of photosynthesis. There is a need to plant more trees around the municipality’. Green places are pivotal for the absorption of GHGs in the atmosphere and managing other environmental problems. However, a small proportion of respondents stated that the growth of the city does not necessarily result in the reduction of green spaces. The reduction of green places is attributed to the perception that the municipality continues to discourage the planting of trees and lawns around their yards. It was discovered that communities in the City of Polokwane are discouraged from planting lawns around their houses because of the persistent drought and water scarcity. The growth of the city has been driven by the municipality through the development of new housing projects and road construction around the City of Polokwane. The developments have resulted in placing more people within the city, at the same time reducing the green spaces.

### The adequacy of infrastructure to manage traffic congestion

**FIGURE 4:**
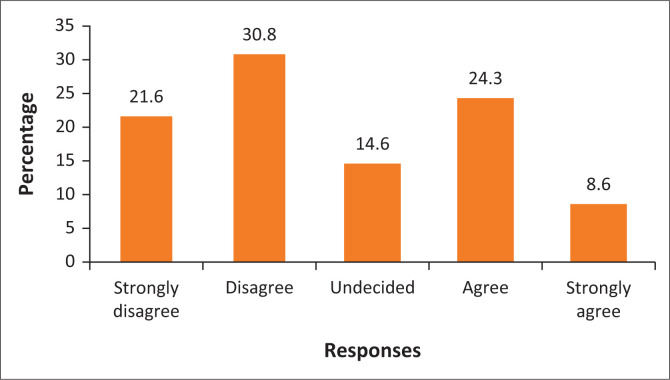
The infrastructure around the city is adequate to control traffic congestion.

Figure 4 shows that 21.6% of the respondents strongly disagreed that there is adequate infrastructure to control traffic congestion around the City of Polokwane. It is indicated in Figure 4 that 30.8% of the respondents disagreed that there is adequate infrastructure to manage traffic congestion. On the other hand, 24.3% and 8.6% agreed and strongly agreed, respectively, that there is adequate road infrastructure around the City of Polokwane. However, only 14.6% of the respondents remained undecided. Serala View and Flora Para have multiple routes into the city, which shows that they are the least affected by traffic congestion. Therefore, Serala View and Flora Park, as middle-income areas, have adequate infrastructure that does not allow traffic congestion. However, a major proportion of the respondents indicated that there is an inadequacy of road infrastructure to manage traffic congestion. Legae la Batho and Emdo Park are confronted with traffic congestion, especially during peak hours (early morning and late in the afternoon). This connotes that the city continues to experience traffic congestion, especially around Legae la Batho and Emdo Park during peak hours, which increases the level of GHGs emissions, hence contributing to climate change in the future. Therefore, the combination of road infrastructure and traffic officers has the potential to address traffic congestion around Polokwane City and helps in the reduction of GHGs emissions. The Director of Development Planning in Polokwane Local Municipality stated that:

‘Polokwane local municipality wants to roll out the Bus Rapid Transit (BRT) transportation which is guided by the *Spatial Planning and Land-Use Management Act*, SPLUMA. The spatial plan promotes a compact city in which concentration regards transport.’ (Respondent C, Director of Development Planning in Polokwane Local Municipality, Interview 08 June 2019)

Despite the challenges such as traffic congestion, Polokwane Local Municipality is making progress towards improving public transport to reduce congestion, which is guided by SPLUMA. The finding is that different areas around the city are affected differently by traffic congestion because of the location and adequacy of road infrastructure. Furthermore, the City of Polokwane, as an economic hub of Limpopo province, provides multiple economic activities such as workplaces and leisure to influence people’s need to travel. This, coupled with the narrow roads in the city, has contributed to the traffic congestion in the City of Polokwane. The few people stating that there is no traffic congestion might be attributed to different socio-economic challenges within the city and their economic activities taking place later in the morning when there is no traffic congestion.

### The collection of refuse by the municipality

**FIGURE 5:**
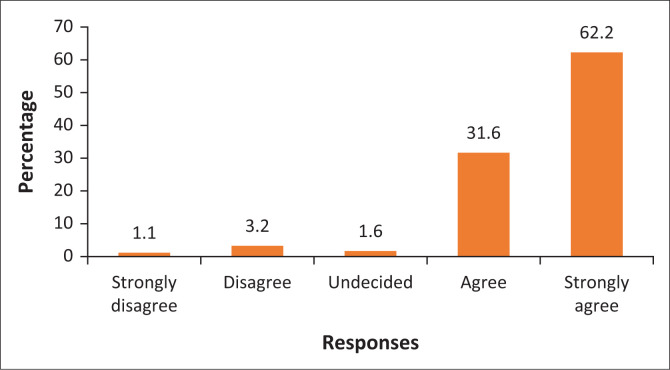
The municipality collects refuse regularly.

The collection of refuse for disposal is very important to reduce emissions such as methane gas. Figure 5 shows that only 1.1% and 3.2% of the respondents strongly disagreed and disagreed, respectively, that waste is regularly collected around the communities. On the other hand, the majority (62.2%) of the respondents strongly agree that waste is collected regularly. Figure 5 demonstrates that 31.9% of the respondents agreed that waste is collected regularly by the municipality. The finding is that Polokwane Local Municipality collects refuse regularly for proper disposal at the municipal landfill. Therefore, proper disposal of waste can reduce the emission of methane, which is generated from waste. Although the Polokwane Local Municipality collects waste around the City of Polokwane, other respondents stated that ‘[s]ome people dispose of their wastes at clearly demarcated areas as illegal dumping sites’. The illegal disposal of waste derails the commitment to reduce GHG emissions and environmental protection around the city. Legae la Batho and Embo Park have problems with illegal dumping sites, as opposed to Flora Park and Serala View. Even though waste is collected weekly in these areas, some people dispose of waste illegally. This is attributed to the fact that at times the collection truck does not collect all the waste, which pushes people to dispose of it illegally. Literature on environmental management states that waste emits a lot of methane, which has the potential to destroy the ozone layer. Effective management of urbanisation to mitigate climate change should be characterised by the safe collection and disposal of waste. Therefore, the finding is that although refuse is collected regularly around the City of Polokwane, some residents continue to dispose of refuse in an unsafe manner, which creates a problem in addressing environmental problems.

### The planning and management of urbanisation

Planning for and management of urbanisation within cities have a multiplicity of complexities to ameliorate various environmental challenges such as climate change and pollution. This is because urbanisation is associated with industrialisation to improve economic growth, which potentially increases electricity consumption, GHGs, waste production and traffic congestion. The Director of Development Planning in Polokwane Local Municipality stated that:

‘The implications of urbanisation need to be put in mind. This is because urbanisation has been demonstrated to have both positive such as industrialisation, economic growth and compact city and negative consequences (GHGs emissions). Some of the strategies implemented are good, but they impact negatively on the institution. The usage of solar, groundwater and gas by households will render the municipality redundant and collapse it.’ (Respondent C, Director of Development Planning in Polokwane Local Municipality, Interview 08 June 2019)

Despite the willingness of people to use gas, groundwater, septic tanks and solar systems within their households, it might negatively impact the municipality at the same time by reducing electricity consumption. The management of urbanisation in mitigating climate change demonstrates the nonlinear properties of the complexity theory. The nonlinearity is based on the fact that the reduction of electricity through these solar systems has unintended consequences on institutions. However, the respondent advocates for the application of a compact city where people can work, play and have leisure time in the same space. This can be used as a way to manage urbanisation, because it promotes an opportunity to walk and use a bicycle to work, as well as the implementation of the BRT.

## Discussion

The policy frameworks such as SDF, IDP and SPLUMA are necessary for the management of urbanisation, but they are not sufficient to ensure that this process is managed effectively. Public participation, integrated transport, stakeholder engagement and promotion of green spaces, solar systems and gas are important to ensure effective planning for urbanisation in an attempt to mitigate climate change. The study found that there is no collaboration between the Polokwane Local Municipality and the communities around during municipal planning. The decline in green spaces, lack of public participation, high consumption of electricity generated from the combustion of fossil fuels and reliance on private cars demonstrate that it is not effectively planned and managed in the City of Polokwane. This is coupled with the increasing traffic congestion within the city, which increases GHGs. The implementation of different modes of transport systems and walkways around the City of Polokwane helps to reduce traffic congestion. Furthermore, the rollout of the BRT (*Leeto la Polokwane*, as it is referred to) provides the potential to increase the usage of public transportation. Quantitative data from the communities provided evidence that the majority of people are not included in municipal planning. Planning and management of urbanisation within the municipality take place in the absence of the community. Therefore, the absence of these factors reveals that the process of urbanisation in the City of Polokwane is not effectively planned and managed.

## Conclusion and recommendations

The planning and management of urbanisation are very complex. The use of integrated transport systems, solar systems and gas, coupled with effective policy frameworks, are important components to effective manage urbanisation. The rollout of the BRT system provides an important trajectory towards reducing traffic congestion. Therefore, the proper management of urbanisation through the BRT system is significant in reducing traffic congestion, which has the potential to reduce the amount of GHG emissions. This is very important for the introduction of an energy mix for climate change mitigation. This energy mix is embodied in the National Development Plan and municipal IDP and connotes the shifting away from overreliance on the burning of fossil fuel for the generation of electricity. It can be recommended that the Polokwane Local Municipality should develop a strategy that will be used to ensure that the community engage and participate in municipal planning systems. Collaborative planning is an important approach that can remove the persisting top-down approach to planning with Polokwane Local Municipality. Furthermore, the plantation of green spaces is a very important environmental management strategy to reduce GHG emissions. Shifting from the heavy reliance on coal or fossil fuels towards the usage of solar systems and gas appliances by households in urban areas will significantly contribute to climate change mitigation. However, this does not mean the abandonment of the usage of electricity within the households. This connotes the implementation of hybrid systems that use electricity, solar systems, batteries and gas appliances.
